# Coagulopathy of hospitalised COVID-19: A Pragmatic Randomised Controlled Trial of Therapeutic Anticoagulation versus Standard Care as a Rapid Response to the COVID-19 Pandemic (RAPID COVID COAG – RAPID Trial): A structured summary of a study protocol for a randomised controlled trial

**DOI:** 10.1186/s13063-021-05076-0

**Published:** 2021-03-10

**Authors:** Michelle Sholzberg, Grace H. Tang, Elnara Negri, Hassan Rahhal, Lisa Baumann Kreuziger, Carlos E. Pompilio, Paula James, Michael Fralick, Musaad AlHamzah, Faris Alomran, Eric Tseng, Gloria Lim, David Lillicrap, Marc Carrier, Fionnuala Ní Áinle, Andrew Beckett, Bruno R. da Costa, Kevin Thorpe, Saskia Middeldorp, Agnes Lee, Mary Cushman, Peter Jüni

**Affiliations:** 1 St. Michael’s Hospital, Li Ka Shing Knowledge Institute, University of Toronto, 30 Bond Street, Room 2-007G Core Lab, Cardinal Carter Wing, Toronto, Ontario M5B-1W8 Canada; 2 St. Michael’s Hospital, University of Toronto, Toronto, Canada; 3 Hospital das Clínicas da Faculdade de Medicina da Universidade de São Paulo, São Paulo, Brazil; 4 Versiti, Medical College of Wisconsin, Milwaukee, USA; 5 Department of Medicine, Queen’s University, Kingston, Canada; 6 Mount Sinai Hospital, University of Toronto, Toronto, Canada; 7 Department of Surgery, King Saud University Medical City, King Saud University, Riyadh, Saudi Arabia; 8 King Faisal Specialist Hospital and Research Centre, Riyadh, Saudi Arabia; 9 Kingston General Hospital, Queen’s University, Kingston, Canada; 10 Department of Medicine, Ottawa Hospital Research Institute at the University of Ottawa, Ottawa, Canada; 11 The Mater Misericordiae University Hospital, School of Medicine, University College Dublin, Dublin, Ireland; 12 Irish Network for VTE Research (INViTE), Dublin, Ireland; 13 St. Michael’s Hospital, Applied Health Research Centre, Li Ka Shing Knowledge Institute, University of Toronto, Toronto, Canada; 14 Amsterdam UMC, University of Amsterdam, Amsterdam, The Netherlands; 15 Vancouver General Hospital, University of British Columbia, Vancouver, Canada; 16 Larner College of Medicine, University of Vermont, Burlington, USA

**Keywords:** COVID-19, anticoagulation, heparin, coagulopathy, randomised controlled trial, and protocol

## Abstract

**Objectives:**

To determine the effect of therapeutic anticoagulation, with low molecular weight heparin (LMWH) or unfractionated heparin (UFH, high dose nomogram), compared to standard care in hospitalized patients admitted for COVID-19 with an elevated D-dimer on the composite outcome of intensive care unit (ICU) admission, non-invasive positive pressure ventilation, invasive mechanical ventilation or death up to 28 days.

**Trial design:**

Open-label, parallel, 1:1, phase 3, 2-arm randomized controlled trial

**Participants:**

The study population includes hospitalized adults admitted for COVID-19 prior to the development of critical illness. Excluded individuals are those where the bleeding risk or risk of transfusion would generally be considered unacceptable, those already therapeutically anticoagulated and those who have already have any component of the primary composite outcome. Participants are recruited from hospital sites in Brazil, Canada, Ireland, Saudi Arabia, United Arab Emirates, and the United States of America.

The inclusion criteria are:

1. Laboratory confirmed COVID-19 (diagnosis of SARS-CoV-2 via reverse transcriptase polymerase chain reaction as per the World Health Organization protocol or by nucleic acid based isothermal amplification) prior to hospital admission OR within first 5 days (i.e. 120 hours) after hospital admission;
2. Admitted to hospital for COVID-19;
3. One D-dimer value above the upper limit of normal (ULN) (within 5 days (i.e. 120 hours) of hospital admission) AND EITHER:
  - D-Dimer ≥2 times ULN OR
  - D-Dimer above ULN and Oxygen saturation ≤ 93% on room air;
4. > 18 years of age;
5. Informed consent from the patient (or legally authorized substitute decision maker).

The exclusion criteria are:

1. pregnancy;
2. hemoglobin <80 g/L in the last 72 hours;
3. platelet count <50 x 10^9^/L in the last 72 hours;
4. known fibrinogen <1.5 g/L (if testing deemed clinically indicated by the treating physician prior to the initiation of anticoagulation);
5. known INR >1.8 (if testing deemed clinically indicated by the treating physician prior to the initiation of anticoagulation);
6. patient already prescribed intermediate dosing of LMWH that cannot be changed (determination of what constitutes an intermediate dose is to be at the discretion of the treating clinician taking the local institutional thromboprophylaxis protocol for high risk patients into consideration);
7. patient already prescribed therapeutic anticoagulation at the time of screening [low or high dose nomogram UFH, LMWH, warfarin, direct oral anticoagulant (any dose of dabigatran, apixaban, rivaroxaban, edoxaban)];
8. patient prescribed dual antiplatelet therapy, when one of the agents cannot be stopped safely;
9. known bleeding within the last 30 days requiring emergency room presentation or hospitalization;
10. known history of a bleeding disorder of an inherited or active acquired bleeding disorder;
11. known history of heparin-induced thrombocytopenia;
12. known allergy to UFH or LMWH;
13. admitted to the intensive care unit at the time of screening;
14. treated with non-invasive positive pressure ventilation or invasive mechanical ventilation at the time of screening;
15. Imminent death according to the judgement of the most responsible physician;
16. enrollment in another clinical trial of antithrombotic therapy involving hospitalized patients.

**Intervention and comparator:**

Intervention: Therapeutic dose of LMWH (dalteparin, enoxaparin, tinzaparin) or high dose nomogram of UFH. The choice of LMWH versus UFH will be at the clinician’s discretion and dependent on local institutional supply.

Comparator: Standard care [thromboprophylactic doses of LMWH (dalteparin, enoxaparin, tinzaparin, fondaparinux)] or UFH. Administration of LMWH, UFH or fondaparinux at thromboprophylactic doses for acutely ill hospitalized medical patients, in the absence of contraindication, is generally considered standard care.

**Main outcomes:**

The primary composite outcome of ICU admission, non-invasive positive pressure ventilation, invasive mechanical ventilation or death at 28 days.

Secondary outcomes include (evaluated up to day 28):

1. All-cause death
2. Composite of ICU admission or all-cause death
3. Composite of mechanical ventilation or all-cause death
4. Major bleeding as defined by the ISTH Scientific and Standardization Committee (ISTH-SSC) recommendation;
5. Red blood cell transfusion (>1 unit);
6. Transfusion of platelets, frozen plasma, prothrombin complex concentrate, cryoprecipitate and/or fibrinogen concentrate;
7. Renal replacement therapy;
8. Hospital-free days alive;
9. ICU-free days alive;
10. Ventilator-free days alive;
11. Organ support-free days alive;
12. Venous thromboembolism (defined as symptomatic or incidental, suspected or confirmed via diagnostic imaging and/or electrocardiogram where appropriate);
13. Arterial thromboembolism (defined as suspected or confirmed via diagnostic imaging and/or electrocardiogram where appropriate);
14. Heparin induced thrombocytopenia;
15. Trajectories of COVID-19 disease-related coagulation and inflammatory biomarkers.

**Randomisation:**

Randomisation will be stratified by site and age (>65 versus ≤65 years) using a 1:1 computer-generated random allocation sequence with variable block sizes. Randomization will occur within the first 5 days (i.e. 120 hours) of participant hospital admission. However, it is recommended that randomization occurs as early as possible after hospital admission. Central randomization using an interactive web response system will ensure allocation concealment.

**Blinding (masking):**

No blinding involved. This is an open-label trial.

**Numbers to be randomised (sample size):**

462 patients (231 per group) are needed to detect a 15% risk difference, from 50% in the control group to 35% in the experimental group, with power of 90% at a two-sided alpha of 0.05.

**Trial Status:**

Protocol Version Number 1.4. Recruitment began on May 11^th^, 2020. Recruitment is expected to be completed March 2022. Recruitment is ongoing.

**Trial registration:**

ClinicalTrials.gov Identifier: NCT04362085

Date of Trial Registration: April 24, 2020

**Full protocol:**

The full protocol is attached as an additional file, accessible from the Trials website (Additional file 1). In the interest of expediting dissemination of this material, the familiar formatting has been eliminated; this Letter serves as a summary of the key elements of the full protocol.

**Supplementary Information:**

The online version contains supplementary material available at 10.1186/s13063-021-05076-0.

## Supplementary Information

**Additional file 1.**

## Data Availability

Data are stored at the Applied Health Research Centre in Toronto, Canada. Co-principal investigators will have access to the data as will co-investigators per coordination site and study site specific agreements with St. Michael’s Hospital, Toronto, Canada.

