# Supplementary material for: Coagulopathy of hospitalised COVID-19: A Pragmatic Randomised Controlled Trial of Therapeutic Anticoagulation versus Standard Care as a Rapid Response to the COVID-19 Pandemic (RAPID COVID COAG – RAPID Trial): A structured summary of a study protocol for a randomised controlled trial
Source: Trials. 2021 Mar 10;22:202. doi: 10.1186/s13063-021-05076-0 (PMC7943934; doi:10.1186/s13063-021-05076-0)
Supplement: Supplementary file 1 — Additional file 1. [file 13063_2021_5076_MOESM1_ESM.docx]

**Coagulopathy of COVID-19: A Pragmatic Randomized Controlled Trial of Therapeutic Anticoagulation versus Standard Care as a Rapid Response to the COVID-19 Pandemic (RAPID COVID COAG)**

**Co-Principal Investigators:**

Dr. Michelle Sholzberg, St. Michael’s Hospital, Li Ka Shing Knowledge Institute, University of Toronto

Dr. Peter Jüni, St. Michael’s Hospital, Li Ka Shing Knowledge Institute, University of Toronto

Dr. Mary Cushman, University of Vermont Medical Center

**Steering Commitee:**

Dr. Musaad Alhamzah, King Saud University Medical City, King Saud University

Dr. Lisa Baumann Kreuziger, Versiti, Medical College of Wisconsin

Dr. Andrew Beckett, St. Michael’s Hospital, University of Toronto

Dr. Marc Carrier, The Ottawa Hospital, University of Ottawa

Dr. Bruno da Costa, St. Michael’s Hospital, Applied Health Research Centre (AHRC), Li Ka Shing Knowledge Institute, University of Toronto

Dr. Mike Fralick, Mount Sinai Hospital, University of Toronto

Dr. Paula James, Kingston General Hospital, Queen’s University

Dr. Agnes Lee, Vancouver General Hospital, University of British Columbia

Dr. David Lillicrap, Kingston General Hospital, Queen’s University

Dr. Saskia Middeldorp, Amsterdam UMC, University of Amsterdam, The Netherlands.

Dr. Fionnuala Ní Áinle, Mater Misericordiae University Hospital,University College Dublin, Ireland

Dr. Elnara Negri, Hospital das Clínicas da Faculdade de Medicina da Universidade de São Paulo

Ms. Grace Tang, St. Michael’s Hospital, University of Toronto

Dr. Kevin E. Thorpe, St. Michael’s Hospital, Applied Health Research Centre (AHRC), Li Ka Shing Knowledge Institute, University of Toronto

**Study Summary**

Coagulopathy of COVID-19 afflicts approximately 20% of patients with severe COVID-19 and is associated with need for critical care and death. COVID-19 coagulopathy is characterized by elevated D-dimer, an indicator of fibrin formation and clot lysis, and a mildly prolonged prothrombin time, suggestive of coagulation consumption. To date, it seems that COVID-19 coagulopathy manifests with thromboembolism, thus anticoagulation may be of benefit. We propose to conduct a parallel pragmatic multi-centre open-label randomized controlled trial to determine the effect of therapeutic anticoagulation compared to standard care in hospitalized patients admitted for COVID-19 with an elevated D-dimer.

**Current Protocol Version:** Version 1.4 – 13 Oct 2020

**Clinical trial registration:** <https://clinicaltrials.gov/ct2/show/NCT04362085>

**Sponsor:**

Unity Health Toronto – St. Michael’s Hospital

30 Bond St, Toronto, ON M5B 1W8

Canada

**GCP Statement**

This study document is the protocol for research involving human participants. This study is to be conducted according to Canadian and international standards, and in compliance with the protocol, International Conference on Harmonisation Good Clinical Practice E6 (ICH-GCP), World Medical Association Declaration of Helsinki: Ethical Principles for Medical Research Involving Human Participants, and all applicable regulatory and institutional requirements and research policies.

**Confidentiality Statement**

This clinical study protocol contains information which is of a confidential, trade-secret or proprietary nature. The protocol is for the use of Sponsor-Investigators and their designated representatives participating in the investigational trial. It is not to be disclosed to any other person or party without the prior written approval of Sponsor-Investigator.

Table of Contents

[Protocol Synopsis 5](#_Toc60218199)

[1.0 Background 8](#_Toc60218200)

[2.0 Objectives 9](#_Toc60218201)

[3.0 Study Design 10](#_Toc60218202)

[4.0 Study Population 11](#_Toc60218203)

[4.1 Informed Consent Process 12](#_Toc60218204)

[4.2 Treatment Allocation 12](#_Toc60218205)

[5.0 Description of Treatment vs. Standard Care 12](#_Toc60218206)

[5.1 Therapeutic Anticoagulation 12](#_Toc60218207)

[Table 1. Therapeutic Dose Anticoagulation 13](#_Toc60218208)

[Table 2. Investigational Products Rationale for Inclusion 14](#_Toc60218209)

[5.2 Standard Care 15](#_Toc60218210)

[Table 3. Standard care (low dose, prophylactic) anticoagulation 16](#_Toc60218211)

[Table 4. Formulations of Investigational Products and Standard Care 17](#_Toc60218212)

[6.0 Study Procedures and Evaluations 19](#_Toc60218213)

[6.1 Bloodwork 19](#_Toc60218214)

[6.2 Optional Biobanking 19](#_Toc60218215)

[6.3 Endpoint Assessment 20](#_Toc60218216)

[6.4 Duration of the study 21](#_Toc60218217)

[7.0 Statistical Plan 21](#_Toc60218218)

[7.1 Sample Size Calculation 21](#_Toc60218219)

[7.2 Statistical Analysis 21](#_Toc60218220)

[7.3 Interim Analysis 22](#_Toc60218221)

[8.0 Safety and Adverse Events 22](#_Toc60218222)

[8.1 Definitions 24](#_Toc60218223)

[8.2 Reporting of Serious Adverse Events 26](#_Toc60218224)

[8.3 Monitoring of AEs/SAEs 27](#_Toc60218225)

[8.4 Treatment Discontinuation 27](#_Toc60218226)

[8.5 Premature Study Withdrawal of an Individual Participant 28](#_Toc60218227)

[8.6 Protocol Violations/Deviations 28](#_Toc60218228)

[9.0 Data Handling and Record Keeping 28](#_Toc60218229)

[9.1 Data Management Responsibilities 28](#_Toc60218230)

[9.2 Confidentiality 29](#_Toc60218231)

[9.3 Record Retention 30](#_Toc60218232)

[10.0 Quality Control and Quality Assurance 30](#_Toc60218233)

[10.1 Study Monitoring Plan 30](#_Toc60218234)

[10.2 Role of the Data Safety Monitoring Board 30](#_Toc60218235)

[10.3 Ethics and Research Governance: 31](#_Toc60218236)

[10.4 Feasibility 31](#_Toc60218237)

[10.5 Study Product Supply and Accountability 32](#_Toc60218238)

[11.0 Study Significance 32](#_Toc60218239)

[12.0 Disclosure and Publication Policy 32](#_Toc60218240)

[12.1 Publication of Study Results 32](#_Toc60218241)

[12.2 Data Sharing for Secondary Research 33](#_Toc60218242)

[12.3 Conflict of interest 33](#_Toc60218243)

[Table 5. Study Schedule of Events 34](#_Toc60218244)

[13.0 References 37](#_Toc60218245)

[Appendix 1. Racial and Ethnic Categories and Definitions 39](#_Toc60218246)

[Appendix 2. Requirement for prohibited concomitant medications in the therapeutic anticoagulation (experimental arm) 40](#_Toc60218247)

[Appendix 3. Fraction of Inspired Oxygen (FiO2) for Various Oxygen Delivery Devices 41](#_Toc60218248)

[Appendix 4. Outcome Definition for Organ-support Free Days 43](#_Toc60218249)

# Protocol Synopsis

| Full Title | **Coagulopathy of COVID-19: A Pragmatic Randomized Controlled Trial of Therapeutic Anticoagulation versus Standard Care as a Rapid Response to the COVID-19 Pandemic** |
| --- | --- |
| Short Title | **RAPID COVID COAG** |
| Version Number and Date | Version 1.4, 13 Oct 2020 |
| Clinical Phase | III |
| Study Duration | Enrollment period: April 24, 2020 to March 10, 2022 |
|  | Study period: April 24, 2020 to April 24, 2022 |
| Co-Principal Investigators | Dr. Michelle Sholzberg, Dr. Peter Jüni and Dr. Mary Cushman |
| Study Design | Open-label, parallel, 2-arm randomized controlled trial |
| Primary Objective | To determine the effect of therapeutic anticoagulation, with low molecular weight heparin or unfractionated heparin (high dose nomogram), compared to standard care in hospitalized patients admitted for COVID-19 with an elevated D-dimer on the composite outcome of intensive care unit (ICU) admission, non-invasive positive pressure ventilation, invasive mechanical ventilation or death at 28 days. |
| Secondary Objectives | To compare the following secondary outcomes between study arms up to day 28:   1. All-cause death; 2. Composite of ICU admission or all-cause death; 3. Composite of mechanical ventilation or all-cause death; 4. Major bleeding as defined by the ISTH Scientific and Standardization Committee (ISTH-SSC) recommendation; 5. Red blood cell transfusion (>1 unit); 6. Transfusion of platelets, frozen plasma, prothrombin complex concentrate, cryoprecipitate and/or fibrinogen concentrate; 7. Renal replacement therapy; 8. Hospital-free days alive; 9. ICU-free days alive; 10. Ventilator-free days alive; 11. Organ support-free days alive; 12. Venous thromboembolism (defined as symptomatic or incidental, suspected or confirmed via diagnostic imaging and/or electrocardiogram where appropriate); 13. Arterial thromboembolism (defined as suspected or confirmed via diagnostic imaging and/or electrocardiogram where appropriate); 14. Heparin induced thrombocytopenia; 15. Trajectories of COVID-19 disease-related biomarkers. |
| Sample Size | 462 patients (231 per group). |
| Randomization | 1:1 randomization, centralized web-based randomization. |
| Study Population | The study population includes hospitalized adults admitted for COVID-19 prior to the development of critical illness. We have excluded individuals where the bleeding risk or risk of transfusion would generally be considered unacceptable, those already therapeutically anticoagulated and those who would have already met components of our primary outcome. |
| Investigational Product Description | Therapeutic dose low molecular weight heparin (Dalteparin, Enoxaparin, Tinzaparin) or high dose nomogram of unfractionated heparin. |
| Control | Standard care (thromboprophylactic doses of low molecular weight heparin (Dalteparin, Enoxaparin, Tinzaparin, Fondaparinux) or unfractionated heparin. |
| Duration of Treatment | Therapeutic anticoagulation will be administered until discharged from hospital, 28 days or death. If the patient is admitted to the ICU or requiring ventilatory support, we recommend continuation of the allocated treatment as long as the treating physician is in agreement. |
| Outcome Measures | Primary: composite outcome of intensive care unit (ICU) admission, non-invasive positive pressure ventilation, invasive mechanical ventilation or death at 28 days. |
|  | Secondary (evaluated up to day 28):   1. All-cause death 2. Composite of ICU admission or all-cause death 3. Composite of mechanical ventilation or all-cause death 4. Major bleeding as defined by the ISTH Scientific and Standardization Committee (ISTH-SSC) recommendation; 5. Red blood cell transfusion (>1 unit); 6. Transfusion of platelets, frozen plasma, prothrombin complex concentrate, cryoprecipitate and/or fibrinogen concentrate; 7. Renal replacement therapy; 8. Hospital-free days alive; 9. ICU-free days alive; 10. Ventilator-free days alive; 11. Organ support-free days alive; 12. Venous thromboembolism (defined as symptomatic or incidental, suspected or confirmed via diagnostic imaging and/or electrocardiogram where appropriate); 13. Arterial thromboembolism (defined as suspected or confirmed via diagnostic imaging and/or electrocardiogram where appropriate); 14. Heparin induced thrombocytopenia; 15. Trajectories of COVID-19 disease-related biomarkers. |
| Statistical Analysis | Primary analyses will be by in the intention-to-treat population of all randomized patients in accordance with the originally allocated intervention. We will conduct a chi-square test for the main analysis of the primary outcome and use a generalized linear regression model with binary distribution and identity link to derive risk difference with 95% confidence intervals. |

1.0 Background
Coagulopathy of COVID-19 appears to afflict approximately 20% of patients with severe COVID-19 and is associated with need for critical care and death (1–3). COVID-19 coagulopathy is characterized by elevated D-dimer, an indicator of fibrin formation and clot lysis, and a mildly prolonged prothrombin time (PT), suggestive of coagulation consumption (2–6). In the largest study to date, Guan et al. described 560 patients with COVID-19 where D-dimer was measured and was elevated D-dimer (>0.5 mg/L) in 60% of those with severe illness (1,4). In fact, Zhou et al. noted that a D-dimer concentration greater than 1 µg/mL was associated with mortality in 191 patients with COVID-19 (odds ratio [OR] 18.42, 95%CI: 2.64-128.55, p =0.0033) (5). In a study by Tang et al., where they specifically examined laboratory coagulation parameters in 183 patients, they found that non-survivors of COVID-19 had a D-dimer of 2.12µg/mL (range: 0.77-5.27µg/mL) compared to survivors with 0.61µg/mL (range: 0.61-1.29 µg/mL) (3). Low fibrinogen has been more rarely described with severe COVID-19 and seems to occur later in the course of hospitalization (3). Interestingly, thrombocytopenia, typically associated with sepsis mortality, is not a consistent manifestation of COVID-19 coagulopathy and does not appear to correlate with severity of illness. In fact, very few patients have clinically relevant thrombocytopenia (i.e. a platelet count below 50 x 10^9^/L) (6,7).

In the published literature thus far, it seems that COVID-19 coagulopathy may manifest with thrombosis of the pulmonary microvasculature with secondary pulmonary hemorrhagic infarction and necrosis(2,8). Moreover, there are numerous animal models of acute lung injury describing the benefit of anticoagulation (9,10). Tang et al. found that in patients with severe COVID-19 and laboratory evidence of early disseminated intravascular coagulation (DIC), using the sepsis-induced coagulopathy (SIC) score, had a lower risk of death when treated with low molecular weight heparin (LMWH) or unfractionated heparin (UFH) at thromboprophylactic doses for 7 days or longer (2). In fact, the authors found that 28-day mortality of heparin users were lower than non-users in patients with SIC scores > 4 (40.0% vs. 64.2%, p= 0.029), or D-dimer > 6 times the upper limit of normal (ULN) (32.8% vs. 52.4%, p=0.017)(2). However, this study was limited by its retrospective nature and did not account for other treatments that were provided. Moreover, a recent study by Klok et al. of 184 critically ill patients with COVID-19, reported a 31% incidence of arterial and venous thromboembolism despite pharmacological thromboprophylaxis (11). To the best of our knowledge, there have been very few published reports of clinically significant hemorrhage as a clinical manifestation of COVID-19 coagulopathy (12). Thus, anticoagulation with heparin-based therapy, whether unfractionated or low molecular weight (Dalteparin, Enoxaparin, Tinzaparin), for severe COVID-19 may theoretically be of benefit (9,10).

Currently, our group is conducting a systematic review and meta-analysis to describe hemostatic parameters and relevant clinical outcomes (e.g. thromboembolism, bleeding, death) among patients with COVID-19 (PROSPERO: (13)). To date, there are 62 full-text articles that have met our inclusion criteria and data extraction is ongoing.

COVID-19 coagulopathy is such an increasingly recognized manifestation of severe illness, that the International Society on Thrombosis and Haemostasis (ISTH) recently published a guidance document on its management despite the current paucity of evidence (4). In addition, anticoagulation for patients with severe COVID-19 has been recommended by a Chinese expert consensus statement, however without proper evidence to support its efficacy (14). Moreover, there is evidence that D-dimer levels and markers of inflammation vary at baseline according to race/ethnicity with more adverse levels in Hispanic and especially African-American persons (15,16). This, combined with the observation that individuals of minority groups are more likely to experience severe and fatal COVID-19 highlights the need to evaluate whether the effect of anticoagulation, and COVID-19 coagulopathy biomarkers differ by race/ethnicity.

We believe that the current knowledge of COVID-19 coagulopathy provides rationale for a pragmatic randomized controlled trial (RCT) to evaluate the effectiveness of therapeutic anticoagulation with LMWH or UFH (high dose nomogram) in patients with severe COVID-19 and an elevated D-dimer compared to standard care, which typically includes LMWH or UFH at thromboprophylactic doses.

2.0 Objectives  **Primary objective**:

To determine the effect of therapeutic anticoagulation, with LMWH or UFH (high dose nomogram), compared to standard care in hospitalized patients admitted for COVID-19 with an elevated D-dimer on the composite outcome of intensive care unit (ICU) admission, non-invasive positive pressure ventilation, invasive mechanical ventilation or death at 28 days.

S**econdary objectives:**

To determine the effect of therapeutic anticoagulation, with LMWH or UFH (high dose nomogram), compared to standard care in hospitalized patients admitted for COVID-19 with an elevated D-dimer, up to day 28, on:

1. All-cause death;
2. Composite of ICU admission or all-cause death;
3. Composite of mechanical ventilation or all-cause death;
4. Major bleeding as defined by the ISTH Scientific and Standardization Committee (ISTH-SSC) recommendation;
5. Red blood cell transfusion (>1 unit);
6. Transfusion of platelets, frozen plasma, prothrombin complex concentrate, cryoprecipitate and/or fibrinogen concentrate;
7. Renal replacement therapy defined as continuous renal replacement therapy {CRRT} or intermittent hemodialysis {IHD};
8. Hospital-free days alive;
9. ICU-free days alive;
10. Ventilator-free days alive;
11. Organ support-free days alive (see Appendix 4 for definition);
12. Venous thromboembolism (defined as symptomatic or incidental, suspected or confirmed via diagnostic imaging and/or electrocardiogram where appropriate recognizing that access to diagnostic imaging may be limited in the midst of the COVID-19 pandemic. Confirmatory testing at a later date once exposure risk/personal protective utilization is not an issue is encouraged);
13. Arterial thromboembolism (defined as suspected or confirmed via diagnostic imaging and/or electrocardiogram where appropriate recognizing that access to diagnostic imaging may be limited in the midst of the COVID-19 pandemic. Confirmatory testing at a later date once exposure risk/personal protective utilization is not an issue is encouraged);
14. Heparin induced thrombocytopenia;
15. Trajectories of COVID-19 disease-related biomarkers.

**Tertiary Objective**

1. To explore whether the above outcomes differ by age, sex, diabetes mellitus, coronary artery disease, hypertension, race/ethnicity, D-dimer levels and hypoxia.

The use of BIPAP or CPAP at night or when sleeping for sleep apnea is not considered non-invasive mechanical ventilation or organ support for the purpose of this trial.

3.0 Study Design
We propose to conduct a 2-arm, parallel, pragmatic, multi-centre, open-label randomized controlled trial to determine the effect of therapeutic anticoagulation on the composite outcome of ICU admission, invasive mechanical ventilation or death in hospitalized patients admitted for COVID-19. Eligible participants will be randomized to one of two treatment regimens, receiving either therapeutic anticoagulation or standard care until discharged from hospital, death or day 28. If the patient is admitted to the ICU or requiring ventilatory support, we recommend continuation of the allocated treatment as long as the treating physician is in agreement. Randomization will be stratified by site and age (>65 versus ≤65 years) using a 1:1 computer-generated random allocation sequence with variable block sizes. Randomization will occur within the first 5 days (i.e. 120 hours) of participant hospital admission. However, it is recommended that randomization occurs as early as possible after hospital admission. Central randomization using an interactive web response system will ensure allocation concealment. This is an open label study. Blinding of participants, clinical research staff, and clinicians is not possible due to the nature of the intervention. However, the biostatisticians will be blinded at the data analysis phase. Given the objective nature of the primary outcome and the need for pragmatic approaches for this rapid response trial, an independent, blind clinical events committee is not planned (17). See Figure 1 for study schematic.


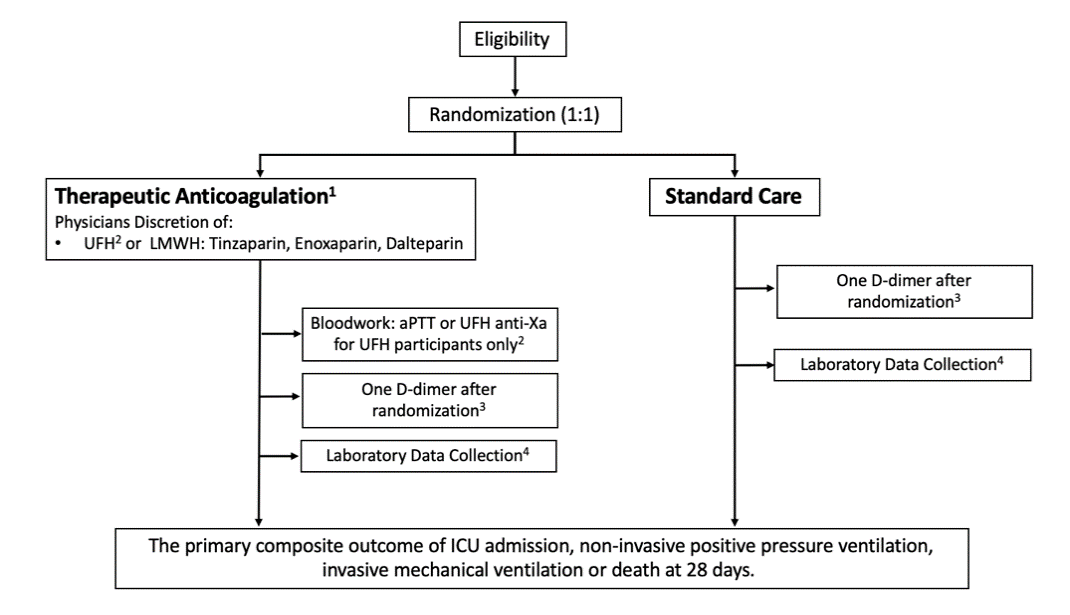


**Figure 1. RAPID COVID-COAG Study Schematic**

^1^Administer until hospital discharge, death or day 28, if the patient is admitted to the ICU or requiring ventilatory support, we recommend continuation of the allocated treatment as long as the treating physician is in agreement;

^2^For participants treated with UFH: aPTT or UFH anti-Xa will be drawn according to local institutional UFH nomogram protocol guidance;

^3^a single D-dimer test (if not collected through standard of care) on day 2 after randomization (+/- 24 hours) will be collected for participants in both study arms (considering the day of randomization as day 1).

^4^All laboratory results, when available, will be collected from standard of care from admission to hospital discharge, death or 28 days from patient chart

4.0 Study Population
The study population includes hospitalized adults admitted for COVID-19 prior to the development of critical illness. We have excluded individuals where the bleeding risk or risk of transfusion would generally be considered unacceptable, those already therapeutically anticoagulated and those who would have already met components of our primary outcome. Participants are recruited from hospital sites in Argentina, Brazil, Canada, Ireland, Saudi Arabia, United Arab Emirates, and the United States of America.

The inclusion criteria are:

1. Laboratory confirmed COVID-19 (diagnosis of SARS-CoV-2 via reverse transcriptase polymerase chain reaction as per the World Health Organization protocol or by nucleic acid based isothermal amplification). Positive test prior to hospital admission **OR** within first 5 days (i.e. 120 hours) after hospital admission;
2. Admitted to hospital for COVID-19;
3. One D-dimer value above ULN (within 5 days (i.e. 120 hours) of hospital admission) **AND** **EITHER**:
   1. D-Dimer ≥2 times ULN **OR**
   2. D-Dimer above ULN and Oxygen saturation ≤ 93% on room air;
4. > 18 years of age;
5. Informed consent from the patient (or legally authorized substitute decision maker).

The exclusion criteria are:

1. pregnancy;
2. hemoglobin <80 g/L in the last 72 hours;
3. platelet count <50 x 10^9^/L in the last 72 hours;
4. known fibrinogen <1.5 g/L (if testing deemed clinically indicated by the treating physician prior to the initiation of anticoagulation);
5. known INR >1.8 (if testing deemed clinically indicated by the treating physician prior to the initiation of anticoagulation);
6. patient already on intermediate dosing of LMWH that cannot be changed (determination of what constitutes an intermediate dose is to be at the discretion of the treating clinician taking the local institutional thromboprophylaxis protocol for high risk patients into consideration);
7. patient already on therapeutic anticoagulation at the time of screening (low or high dose nomogram UFH, LMWH, warfarin, direct oral anticoagulant (any dose of dabigatran, apixaban, rivaroxaban, edoxaban);
8. patient on dual antiplatelet therapy, when one of the agents cannot be stopped safely;
9. known bleeding within the last 30 days requiring emergency room presentation or hospitalization;
10. known history of a bleeding disorder of an inherited or active acquired bleeding disorder;
11. known history of heparin-induced thrombocytopenia;
12. known allergy to UFH or LMWH;
13. admitted to the intensive care unit at the time of screening;
14. treated with non-invasive positive pressure ventilation or invasive mechanical ventilation at the time of screening (of note: high flow oxygen delivery via nasal cannula is acceptable and is not an exclusion criterion);
15. Imminent death according to the judgement of the most responsible physician;
16. enrollment in another clinical trial of antithrombotic therapy involving pre-intensive care unit hospitalized patients.

## 4.1 Informed Consent Process

Potential eligible patients will be confirmed with both the most responsible physician (MRP) and the site investigator within the first 5 days (i.e. 120 hours) of hospital admission. Eligible patients will be consented by a research coordinator or health care worker (other than the MRP) within the first 5 days (i.e. 120 hours) of hospital admission using remote methods (such as telephone consent or eConsent) to obtain verbal or written consent to minimize the risk of COVID-19 exposure, conserve the use of personal protective equipment and prevent COVID-19 research document contamination. Informed consent is to be obtained directly from the patient or from their legally authorized substitute decision maker (applies to initial consent and ongoing consent, should the participant lose capacity during the trial). Capacity will be assessed by the circle of care. Also, where possible, to minimize the burden to the patient, patients may be consented to more than one COVID-19-related clinical trial at a time.

We will follow Health Canada’s Management of clinical trials during the COVID-19 pandemic: Notice to Clinical Trial Sponsors.

## 4.2 Treatment Allocation

PI/Co-I will determine eligibility prior to notifying the research coordinator by phone and email to begin randomization process. Central randomization will occur using an interactive web response system. This is an open label study thus once a participant is randomized, a research coordinator will document the treatment allocation in the case report form and notify the MRP and study team by email and phone of the patient’s study treatment allocation (therapeutic or standard care arm). The MRP will then order the study drug in the patient’s medical chart and notify the study team. The research coordinator will then email or fax research pharmacy the order. Research pharmacy will follow their SOP to dispense study drug and study drug will be dispensed via research pharmacy to the treating medical team.

# 5.0 Description of Treatment vs. Standard Care

## 5.1 Therapeutic Anticoagulation

Therapeutic anticoagulation with LMWH or UFH (high dose nomogram). The choice of LMWH versus UFH will be at the clinician’s discretion and dependent on local institutional supply. LMWH options are described in Table 1 (18–20). UFH will be administered using a weight-based nomogram (bolus plus continuous infusion) with activated partial thromboplastin time (aPTT) or UFH anti-Xa titration according to the center-specific institutional protocols as per venous thromboembolism treatment (i.e. high dose nomogram). UFH anti-Xa titration is preferred over aPTT if available as achieving a therapeutic aPTT may be challenging in patients with a pro-inflammatory state such as COVID-19. Therapeutic anticoagulation will be administered until hospital discharge, death or day 28. If the patient is admitted to the ICU or requiring ventilatory support (i.e. patient reaches a component of the primary outcome), we recommend continuation of the allocated treatment as long as the treating physician is in agreement.

# Table 1. Therapeutic Dose Anticoagulation

Any of the following strategies may be used for high-dose anticoagulation:

| CrCl | BMI | Enoxaparin | Dalteparin | Tinzaparin | UFH |
| --- | --- | --- | --- | --- | --- |
| ≥30 | <40 | 1 mg/kg SC q12h OR  1.5 SC mg/kg q24h | 200 units/kg SC q24h OR  100 IU/kg SC q12h | 175 U/kg SC q24h | IV bolus, with continuous infusion to titrate to institution specific anti-Xa or aPTT values* |
|  | ≥40 | 1 mg/kg q12h^&^ | 100 units/kg SC q12h^&^ | 175 U/kg SC daily^&^ |  |
| <30 | <40 | UFH IV bolus, with continuous infusion to titrate to institution specific anti-Xa or aPTT values* or LMWH per hospital protocol taking BMI into consideration as above | | | |
|  | ≥40 |  |  |  |  |

Abbreviations: CrCl = creatinine clearance; BMI = body mass index; * Initial bolus dose determined by sites, encouraging use of dosing algorithm designed for treatment of VTE. UFH anti‐Xa titration is preferred over aPTT if available as achieving a therapeutic aPTT may be challenging in patients with a pro‐inflammatory state such as COVID‐19.

^&^For patients with BMI above 40, measurement of anti-Xa to confirm therapeutic effect can be used.

Table 2 outlines the investigational products chosen for this study, mechanism of action and rationale for use.

# Table 2. Investigational Products Rationale for Inclusion

| Drug | Mechanism of Action | Route | Rationale for Inclusion |
| --- | --- | --- | --- |
| Unfractionated heparin | Indirect anticoagulant that forms a complex with antithrombin enhancing the inhibition of several activated blood coagulation factors: thrombin (factor IIa), factor IXa, Xa, XIa and XIIa. | Intravenous (IV)  Subcutaneous (SC) | Considered standard care for both prophylactic and therapeutic dose anticoagulation for acutely ill hospitalized medical patients therefore, included as an option in both the standard care (control) arm and therapeutic anticoagulation (experimental) arm. |
| Dalteparin | Indirect anticoagulant that forms a complex with antithrombin enhancing the inhibition of coagulation factor Xa and thrombin (factor IIa). | SC | Considered standard care for both prophylactic and therapeutic dose anticoagulation for acutely ill hospitalized medical patients therefore, included as an option in both the standard care (control) arm and therapeutic anticoagulation (experimental) arm. |
| Enoxaparin | Indirect anticoagulant that forms a complex with antithrombin enhancing the inhibition of coagulation factor Xa and thrombin (factor IIa). | SC | Considered standard care for both prophylactic and therapeutic dose anticoagulation for acutely ill hospitalized medical patients therefore, included as an option in both the standard care (control) arm and therapeutic anticoagulation (experimental) arm. |
| Tinzaparin | Indirect anticoagulant that forms a complex with antithrombin enhancing the inhibition of coagulation factor Xa and thrombin (factor IIa). | SC | Considered standard care for both prophylactic and therapeutic dose anticoagulation for acutely ill hospitalized medical patients therefore, included as an option in both the standard care (control) arm and therapeutic anticoagulation (experimental) arm. |
| Fondaparinux | Indirect anticoagulant that forms a complex with antithrombin enhancing the inhibition of coagulation factor Xa. | SC | Considered standard care for prophylactic dose anticoagulation for acutely ill hospitalized medical patients therefore, included as an option in the standard care (control) arm but not in the therapeutic anticoagulation (experimental) arm. |

## 5.2 Standard Care

In Canada and the US, administration of LMWH, UFH or fondaparinux at thromboprophylactic doses for acutely ill hospitalized medical patients, in the absence of contraindication, is generally considered standard care. Importantly, the doses of thromboprophylaxis only include those listed below (Table 3) given the current equipoise surrounding the effectiveness of weight-based dose adjusted thromboprophylaxis in acutely ill medical inpatients.

Descriptions of the Investigational Products and Standard Care are provided in Table 3 below.

# Table 3. Standard care (low dose, prophylactic) anticoagulation

Any of the following strategies may be used for low-dose anticoagulation (doses above those listed would not be considered as low dose {prophylactic}. A patient is receiving a lower dose of either LMWH or UFH than listed below, due to extremely low weight/BMI, would still be acceptable and considered as part of standard care {low dose, prophylactic anticoagulation}):

**Prophylactic Dose Anticoagulation**

| CrCl | BMI | Enoxaparin | Dalteparin | Tinzaparin | Fondaparinux | Unfractionated Heparin (UFH) |
| --- | --- | --- | --- | --- | --- | --- |
| ≥30 | <40 | 40 mg SC q24h | 5000 units SC q24h | 4500 U SC q24h | 2.5 mg SC q24h | 5000 units SC q8-12h |
|  | ≥40 | 40 mg SC q12h | 5000 units SC q12h | 9000 (+/- 1000) U SC q24h | not applicable | 7500 units SC q8h |
| <30 | <40 | UFH 5000 units SC q8-12h or LMWH per hospital protocol taking BMI into consideration as above | | | | |
|  | ≥40 | UFH 7500 units SC q8h or LMWH per hospital protocol taking BMI into consideration as above | | | | |

Abbreviations: CrCl = creatinine clearance; BMI = body mass index

Type and doses of thromboprophylactic LMWH or UFH will be recorded in the case report forms. Concomitant medications and co-enrollment of other COVID-19 trials will also be recorded.

Full therapeutic dose anticoagulation (therapeutic dose UFH or LMWH) is permitted as rescue therapy in the event of suspected or confirmed thromboembolism. Additional rescue therapy, in the form of thrombolysis (with tissue plasminogen activator), is also permitted if deemed clinically warranted by the treating clinical team.

Insitutional drug stock will be used, and the institutional pharmacy will be responsible for labelling and distribution. No repackaging of drugs will occur.

# Table 4. Formulations of Investigational Products and Standard Care

| **Common Name** | **Brand Name** | **Formulation** | **Manufacturer** | **DIN(s)** |
| --- | --- | --- | --- | --- |
| Tinzaparin Sodium | Innohep | 10000 Unit / mL,  20000 Unit / mL,  8000 Unit / 0.4 mL,  12000 Unit / 0.6 mL,  16000 Unit / 0.8 mL | Leo Pharma Inc. | 02167840, 02229515, 02229755, 02231478, 02358158, 02358166, 02358174, 02358182, 02429462, 02429470, 02429489 |
| Enoxaparin Sodium | Lovenox | 30 mg / 0.3 mL,  40 mg / 0.4 mL,  60 mg / 0.6 mL,  80 mg / 0.8 mL,  100 mg / mL | Sanofi-Aventis Canada Inc. | 02012472, 02236883, 02378426, 02378434, 02378442 |
| Enoxaparin Sodium | Lovenox HP | 120 mg / 0.8 mL,  150 mg / mL | Sanofi-Aventis Canada Inc. | 02242692, 02378469 |
| Enoxaparin Sodium | Lovenox With Preservative 300mg/3mL | 100 mg / mL | Sanofi-Aventis Canada Inc. | 02236564 |
| Dalteparin Sodium | Fragmin | 2500 Unit / 0.2 mL,  5000 Unit / 0.2 mL,  10000 Unit / mL,  25000 Unit / mL,  7500 Unit / 0.3 mL,  10000 Unit / 0.4 mL,  12500 Unit / 0.5 mL,  15000 Unit / 0.6 mL,  18000 Unit / 0.72 mL,  3500 Unit / 0.28 mL | Pfizer Canada Inc. | 02132621, 02132648, 02132664, 02231171, 02352648, 02352656, 02352664, 02352672,  02352680, 02430789 |
| Heparin Sodium | Heparin Leo | 100 Unit / mL,  1000 Unit / mL | Leo Pharma Inc. | 0727520, 00453811 |
| Heparin Sodium | Heparin Sodium Injection, USP | 1000 Unit / mL,  10000 Unit / mL, | Fresenius Kabi Canada Ltd. | 02264307, 02264315, 02392453 |
| Heparin Sodium | Heparin Sodium Injection USP | 1000 Unit / mL,  10000 Unit / mL | Sandoz Canada Inc. | 02303086, 02303108, 02303094 |
| Heparin Sodium | Heparin Sodium Injection USP | 5000 Unit / 0.5 mL | Sterinova Inc. | 02456958 |
| Heparin Sodium | Heparin Sodium Injection USP | 10000 Unit / ML | Pfizer Canada Inc. | 02382326 |
| Heparin Sodium | Heparin Sodium In 0.9% Sodium Chloride Injection | 2 Unit / mL | Pfizer Canada Inc. | 01990748 |
| Heparin Sodium | Heparin Sodium And 0.9% Sodium Chloride Injection | 2 Unit / mL | Baxter Corporation | 00828750 |
| Heparin Sodium | Heparin Sodium 25000 Units In 5% Dextrose Injection | 10000 Unit / 100 mL | B. Braun Medical Inc. | 02209721 |
| Heparin Sodium | Heparin Sodium 20000 Units In 5% Dextrose Injection | 4000 Unit / 100 mL | B. Braun Medical Inc. | 02209713 |
| Heparin Sodium | Heparin Sodium In 5% Dextrose Injection, 25000 Unit/500mL | 5000 Unit / 100 mL | B. Braun Medical Inc. | 01935941 |
| Fondaparinux Sodium | Arixtra | 2.5 mg / 0.5 mL,  7.5 mg / 0.6 mL, | Aspen Pharmacare Canada Inc. | 02245531, 02258056 |
| Fondaparinux Sodium | Fondaparinux Sodium Injection | 2.5 mg / 0.5 mL,  7.5 mg / 0.6 mL | Dr. Reddy's Laboratories Ltd. | 02406853, 02406896 |

6.0 Study Procedures and Evaluations

Please refer to Table 5 for Schedule of Events.

6.1 Bloodwork
No study specific bloodwork will be ordered aside from a single D-dimer test (if not collected through standard of care) up to and including day 3 after randomization for all participants in both study arms. In those on the active treatment arm who are receiving UFH, the aPTT or UFH anti-Xa will be drawn according to local institutional UFH nomogram protocol guidance. Where possible, any study specific blood work will be timed along with regularly scheduled clinical blood work to minimize exposure and use of personal protective equipment. All laboratory results (see Table 5 for Schedule of Events) will be collected from standard of care from admission to hospital discharge, death or 28 days, where available. Laboratory parameters of interest include complete blood count (CBC), PT, aPTT or anti-Xa for UFH (according to local institutional protocol), anti-Xa for LMWH, fibrinogen, blood group, ferritin, lactate dehydrogenase (LDH), troponin, C-reactive protein (CRP), creatinine, alanine aminotransferase [ALT], heparin-platelet factor 4 immunoassay.

Details regarding institutional-specific D-dimer coagulation instrumentation and assay, as well as local reference intervals, including units of measure, will be collected from each site.

## 6.2 Optional Biobanking

For hospital sites with capable infrastructure, the study team will collect samples for biobanking of plasma and serum to evaluate the pathophysiologic mechanism of COVID-19 coagulopathy, an entity which is poorly understood, the impact of anticoagulation on biomarkers, and role of biomarkers in predicting outcomes. Samples will be used for future measurement of biomarkers of coagulation, inflammation, endothelial activation or other relevant measures important in COVID-19. Informed consent for the biobanking component will be conducted, where applicable. Blood will be collected three times:

- Day 1 (day of randomization, prior to treatment initiation whenever possible): One serum (5 ml), one EDTA plasma (10 ml), and two 4.5 ml citrate (9 ml) tubes. Total blood volume 24 ml (or closest volume according to blood draw tube size availability at sites)
- Day 3 (at 48h +/- 24h) of randomization: One serum (5 ml), one EDTA plasma (4 ml), and one citrate (4.5 ml) tubes. Total blood volume 13.5 ml (or closest volume according to blood draw tube size availability at sites)
- One of, (1) Day 14 or if any of the following occur earlier: (2) day of discharge; or (3) day of removal from study; or (4) day that the patient reaches a component of the primary outcome (i.e. ICU admission, non-invasive/invasive mechanical ventilation). All drawn +/24 hours around each time. Repeat Day 3 collection of one serum (5 ml), one EDTA plasma (4 ml), and one citrate (4.5 ml) tubes. Total blood volume 13.5 ml (or closest volume according to blood draw tube size availability at sites)

If the time window for blood collection is problematic due to logistical considerations, collection of the sample as close as possible (before or after) to the target time window is suggested. Phlebotomy will be timed along with regularly scheduled clinical blood work to minimize exposure and use of personal protective equipment.

Processing and storage of blood samples will follow a simple protocol to aliquot serum, two types of plasma and the cell layer from the EDTA tube will be aliquoted into 6 storage vials. Cryovials and labels will be provided to participating sites by the central biorepository site (see below).

The aliquoted samples will be stored in freezers (at -80°C or colder) in a secure location at the local institution until the COVID-19 pandemic allows for shipping to the central biorepository, the Laboratory for Clinical Biochemistry Research at the University of Vermont (360 South Park Drive, Colchester, VT, 05445, USA). Dr. Mary Cushman’s laboratory will be the custodian of the specimens.

The collection, processing and storage of samples will be outlined in Standard Operating Procedures to ensure consistency in specimen handling and therefore, optimization of their quality. Samples will be used for future measurement of biomarkers of coagulation, inflammation and endothelial activation. The Co-PIs and investigator team will establish a committee to review and approve requests for samples, following procedures established for multi-center research over the past 30 years in Dr. Cushman’s laboratory.

We anticipate use of the samples until they are exhausted, but if all scientific use is completed, samples will be destroyed as per institutional practices, at the discretion of the investigator team. All participants will provide informed consent in accord with established procedures, but if they desire withdrawal of their specimens that can be done. Laboratory staff will have no information on participant identity, other personal health information, or randomized treatment assignment.

6.3 Endpoint Assessment
**Primary outcome (primary endpoint):** The primary composite outcome of ICU admission, non-invasive positive pressure ventilation, invasive mechanical ventilation or death at 28 days.

If a patient is discharged alive before 28 days, vital status will be determined using a telephone follow-up. If a patient was discharged alive on mechanical ventilation (invasive or non-invasive) prior to day 28, a call to the patient or a doctor/nurse from the rehabilitation health facility is needed to confirm ventilation status on day 28 and their last day of mechanical ventilation.

**Secondary outcomes (secondary endpoints), evaluated up to day 28, include:**

1. All-cause death;
2. Composite of ICU admission or all-cause death;
3. Composite of mechanical ventilation or all-cause death;
4. Major bleeding as defined by the ISTH Scientific and Standardization Committee (ISTH-SSC) recommendation (21);
5. Red blood cell transfusion (>1 unit);
6. Transfusion of platelets, frozen plasma, prothrombin complex concentrate, cryoprecipiate and/or fibrinogen concentrate;
7. Renal replacement therapy defined as continuous renal replacement therapy {CRRT} or intermittent hemodialysis {IHD};
8. Hospital-free days alive;
9. ICU-free days alive;
10. Ventilator-free days alive;
11. Organ support-free days alive (see Appendix 4 for definition);
12. Venous thromboembolism (defined as symptomatic or incidental, suspected or confirmed via diagnostic imaging and/or electrocardiogram where appropriate recognizing that access to diagnostic imaging may be limited in the midst of the COVID-19 pandemic, however confirmatory testing at a later date once exposure risk/personal protective utilization is not an issue is encouraged);
13. Arterial thromboembolism (defined as suspected or confirmed via diagnostic imaging and/or electrocardiogram where appropriate recognizing that access to diagnostic imaging may be limited in the midst of the COVID-19 pandemic, however confirmatory testing at a later date once exposure risk/personal protective utilization is not an issue is encouraged);
14. Heparin induced thrombocytopenia;
15. Trajectories of COVID-19 disease-related biomarkers.

Outcome measures will be obtained from participants’ hospital medical records and where applicable through a telephone follow-up, as outlined in Table 5: Study Schedule of Events. The use of BIPAP or CPAP at night or when sleeping for sleep apnea is not considered non-invasive mechanical ventilation or organ support for the purpose of this trial.

6.4 Duration of the study
We expect to complete the study in approximately 24 months (for site activation, enrollment, follow-up and close-out), followed by 0.5 months for completion of analyses.

# 7.0 Statistical Plan

7.1 Sample Size Calculation
462 patients (231 per group) are needed to detect a 15% risk difference, from 50% in the control group to 35% in the experimental group (2), with power of 90% at a two-sided alpha of 0.05. No attrition is expected. This calculation takes 2 interim analyses into account. There is no inflation to account for losses to follow-up because we expect these to be very infrequent, and given the nature of the trial, included patients, and outcomes, we may conclude an absence of the primary outcome in patients discharged alive from hospital before 28 days with missing outcome data at day 28.

7.2 Statistical Analysis

Primary analyses will be by in the intention-to-treat population of all randomized patients in accordance with the originally allocated intervention. We will conduct a chi-square test for the main analysis of the primary outcome and use a generalized linear regression model with binary distribution and identity link to derive risk difference with 95% confidence intervals. We will conduct subgroup analyses accompanied by tests of interaction for the following variables: age, sex, BMI, time from COVID-19 symptom onset, diabetes mellitus, coronary artery disease, hypertension and race/ethnicity. Logistic regression and linear regression will be used to analyse binary and continuous secondary outcomes after adjustment for age (used for stratification of randomization). Secondary outcomes are exploratory and will not be adjusted for multiple comparisons. A per-protocol analysis of the primary outcome will be restricted to the per-protocol population of participants, defined as those who received experimental or control intervention as allocated during the first 48 hours after randomization. The analysis of safety outcomes will be done in both, the intention-to-treat and per-protocol populations.

If an outcome is missing in more than 5% of the patients, in addition to the pre-planned strategy of assuming non-outcome if patients were discharged alive from hospital before 28 days, a complete case analysis, an inverse probability weighted analysis and multiple imputation on outcome will also be conducted. The statistical analysis plan will be finalized prior to study closure without prior inspection of the data. All analyses will be conducted in Stata version 15.1.

## 7.3 Interim Analysis

Interim analyses will be done when approximately 25% and 75% of the originally planned number of participants have reached determination of the primary endpoint. A group sequential design has been employed that applies a one-sided boundary. The boundary is based on a Hwang-Shih-DeCani spending function for efficacy. When approximately 75% of the originally planned number of participants have reached determination of the primary endpoint, we will perform a conditional power analysis.

If the conditional power given the accumulated data is <30% and there is robust evidence of harm – either a relevant increase in the risk of major bleeding in the experimental group and the lower limit of the 95% confidence interval for major bleeding excluding 5% on an absolute risk difference scale; or a relevant increase in the risk of all-cause death in the experimental group and the lower limit of the 95% confidence interval for death excluding 1% on an absolute risk difference scale - there will be a non-binding recommendation to stop the trial. If the conditional power is <30%, but there is no robust evidence of harm, we will complete recruitment as planned. The rationale for this approach is that prevention of death (a component of the primary outcome) overrides short-term safety. If major bleeds lead to bleeding related deaths to such an extent that no mortality benefit is likely to be realized the trial should be stopped.

If the conditional power is ≥30 and <60%, we will complete recruitment as planned. If the conditional power is ≥65% and <80%, there will be a non-binding recommendation to increase the sample size to achieve 80% power, if deemed feasible from a recruitment perspective. If the conditional power is ≥80%, we will complete recruitment as planned, provided that the interim analysis against the one-sided boundary for efficacy was negative.

Safety reviews will be done by the Data Safety and Monitoring Board at the formal interim analyses mentioned above, and also when approximately 10% and 50% of the originally planned number of participants have reached determination of the primary endpoint. The Data Safety and Monitoring Board can recommend termination or changes to the trial at any time if deemed necessary due to safety concerns. When 10%, 25% and 50% of the originally planned number of participants have reached determination of the primary endpoint, this recommendation will be at the discretion of the Data Safety and Monitoring Board. When approximately 75% of the originally planned number of participants have reached determination of the primary endpoint, the Data Safety and Monitoring Board will be required to also take into account the conditional power when doing the safety review (see above).

# 8.0 Safety and Adverse Events

**Safety Assessments**

Study drug may be temporarily held by the treating physician if any of the following are present, although, ultimately this is at the discretion of the treating physician:

1. hemoglobin <80g/L;
2. platelet count <50 x 10^9^/L;
3. known fibrinogen <1.5 g/L;
4. known INR >1.8;
5. aPTT >1.5 ULN (if participant not on intravenous UFH)

Possible anticipated adverse events are those associated with **LMWH** (given subcutaneously) use and include the following:

| **Common (>5%)** | **Uncommon (1-5%)** | **Rare (less than 1%)** |
| --- | --- | --- |
| - Hemorrhage - Local irritation - Erythema - Mild pain | - Urinary Tract Infection - Chest Pain - Headache - Nausea - Back pain - Fever - Pain - Constipation - Rash - Dyspnea - Vomiting - Anemia | - Heparin induced thrombocytopenia - Heparin induced thrombosis - Hepatobiliary disorders - Abdominal Pain - Diarrhea |

Possible anticipated adverse events are those associated with **Unfractionated Heparin** (intravenous) use and include the following:

| **Common (>5%)** | **Uncommon (1-5%)** |
| --- | --- |
| - Hemorrhage - Local irritation - Erythema - Mild pain - Ulceration | - Heparin induced thrombocytopenia - Heparin induced thrombosis |

**Specification of Safety Parameters**
Meeting any of the following components will be considered a SAE **related** to the study drug:

1. Bleeding defined as by the ISTH-SSC recommendation:
   1. Fatal bleeding, and/or
   2. Symptomatic bleeding in critical area or organ (such as intracranial, intraspinal, intraocular, retroperitoneal, intraarticular or pericardial, or intramuscular with compartment syndrome, and/or
   3. Bleeding causing a fall of hemoglobin level of 20g/L or more, or leading to transfusion of two or more units of whole blood or red cells;
2. Death due to hemorrhage;
3. ICU admission due to hemorrhage (e.g. meets ISTH-SSC criteria and requires ICU admission);
4. Prolongation of an existing hospitalization due to hemorrhage;
5. Death due to heparin induced thrombocytopenia and thrombosis (HITT);
6. ICU admission due to HITT;
7. Prolongation of an existing hospitalization due to HITT.

**Methods and Timing for Assessing, Recording, and Analyzing Safety Parameters**

The AE reporting period begins on the day of randomization and ends with the final study (follow-up) visit. Investigators and study staff will assess the occurrence of AEs and SAEs at all subject evaluation time points during the study. Adverse events will be recorded in the subject’s medical records and on applicable AE source documents. Regulatory authorities, REBs and investigators will be notified of SAEs in accordance with applicable requirements.

## 8.1 Definitions

**Adverse Event (AE):** An adverse event (AE) is any symptom, sign, illness or experience that develops or worsens in severity during the course of the study. Intercurrent illnesses or injuries should be regarded as adverse events. In addition, abnormal results of diagnostic procedures are considered to be adverse events if the abnormality:

- results in study withdrawal
- is associated with a serious adverse event
- is associated with clinical signs or symptoms
- leads to additional treatment or to further diagnostic tests
- is considered by the investigator to be of clinical significance

**Adverse Drug Reaction (ADR):** Any response to a drug, biologic, or natural health product which is noxious and unintended, which occurs at doses normally used or tested for the diagnosis, treatment or prevention of a disease or the modification of an organic function. A reaction, as opposed to an adverse event, is characterized by the fact that a causal relationship between the product and the occurrence is suspected (i.e. judged to be at least a reasonably possibility).

**Serious Adverse Event (SAE):**

Adverse events are classified as serious or non-serious. A Serious Adverse Event (SAE) is an event that:

1. results in death,
2. are life-threatening,
3. In-patient hospitalization or requires prolongation of an existing hospitalization,
4. results in persistent for significant disability,
5. is a congenital anomaly or birth defect,
6. an important medical event, which may jeopardize the subject, and may require intervention to prevent one of the other serious outcomes noted above.

Meeting any of the following components will be considered a SAE **related** to the study drug:

1. Bleeding defined as by the ISTH-SSC recommendation:
   1. Fatal bleeding, and/or
   2. Symptomatic bleeding in critical area or organ (such as intracranial, intraspinal, intraocular, retroperitoneal, intraarticular or pericardial, or intramuscular with compartment syndrome, and/or
   3. Bleeding causing a fall of hemoglobin level of 20g/L or more, or leading to transfusion of two or more units of whole blood or red cells;
2. Death due to hemorrhage;
3. ICU admission due to hemorrhage (e.g. meets ISTH-SSC criteria and requires ICU admission);
4. Prolongation of an existing hospitalization due to hemorrhage;
5. Death due to heparin induced thrombocytopenia and thrombosis (HITT);
6. ICU admission due to HITT;
7. Prolongation of an existing hospitalization due to HITT.

**Serious Unexpected Adverse Drug Reaction (SUSAR):** An adverse reaction, the nature or severity of which is not consistent with the applicable product information (e.g. the Investigator’s Brochure for an unapproved investigational product or package insert/summary of product characteristics for an approved product) and also meets the criteria of an SAE (as above).

**Unexpected Adverse Drug Reaction (UADR):** An adverse reaction, the nature or severity of which is not consistent with the applicable product information (e.g. the Investigator’s Brochure for an unapproved investigational product or package insert/summary of product characteristics for an approved product).

**Unanticipated Problem:** Any incident, experience, or that meets all of the following criteria:

- Unexpected (in terms of nature, severity, or frequency) given (a) the research procedures that are described in the protocol-related documents, such as the REB-approved research protocol and informed consent document, or the Investigator Brochure; and (b) the characteristics of the research participant population being studied; and
- Related or possibly related to participation in the research (possibly related means there is a reasonable possibility that the incident, experience, or outcome may have been caused by the study drug or procedures involved in the research); and
- Suggests that the research places research participants or others at a greater risk of harm (including physical, psychological, economic, or social harm) than was previously known or recognized.

**Assessment of Severity**

We will grade events according to the following grading system:

- **1** [mild, transient, not interfering with daily activities},
- **2** [moderate, inconvenient, some interference with daily activities],
- **3** [severe, intolerable, significant interruption with daily activities, requires therapy]) will be utilized to assess severity of AEs.

An unexpected event is when the specificity or severity of the event is not consistent with the package inserts or investigational brochure for the drugs under study.

**Causality:**

The investigator is to assess the causal relation using the following terms:

1. **Related** (reasonable possibility that the incident, experience, or outcome may have been caused by the study drug)
2. **Probable** (reaction that follows a reasonable temporal sequence and response pattern),
3. **Possible** (reaction that follows a reasonable temporal sequence and response pattern but could have been produced by a number of other factors), and
4. **Unlikely** (reaction that does not follow a reasonable temporal sequence or there is another compelling alternative explanation but causation by study drug cannot be entirely ruled out).
5. **Not related (unrelated to the study drug)**

**Identification of Adverse Events**

In general, grade 1 events will not be considered as adverse events.Some participants in this study will have pre-existing medical conditions and those pre-existing conditions will not be considered as adverse events. New events that occur or the worsening in frequency or intensity of pre-existing conditions will be reported as adverse events (Schedule of Events). All reportable events as defined above, determined to be an AE based on physical examination, laboratory findings, or other means will be recorded in the source documents and entered in the eCRF. Each event will be recorded on an AE eCRF starting after first dose of study drug has been delivered. The investigator will provide date of onset and resolution, severity, action(s) taken, changes in study drug dosing, causality to study drug, and outcome.

**Follow-up of AEs**

Any safety event that is identified at the last assessment (or an early termination) must be recorded on the appropriate eCRF with the status of the safety event noted. All serious suspected adverse reactions and serious adverse reactions will be followed until resolution or until the patient is medically stable.

## 8.2 Reporting of Serious Adverse Events

In the event of a serious adverse event that is **related** to the study drug (i.e. therapeutic anticoagulation), a SAE reporting form must be sent to the coordinating centre within 24 hours.

In the event of a serious adverse event that is **not related** to the study drug (i.e. therapeutic anticoagulation), a SAE reporting form must be sent to the study sponsor (St. Michael’s Hospital) within 72 hours.

**Please email all SAE reporting with the subject line: !![SAE ALERT] <SITE> and <PATIENT ID> to #########.**

The research team will be responsible for reporting SAEs to Health Canada on behalf of the Sponsor-Investigator.

Any, serious, related, unexpected (SUSARs), which occur within or outside of Canada will be reported to Health Canada according to the following criteria:

- Where it is neither fatal or life threatening, within 15 days after becoming aware of the information.
- Where it is fatal or life threatening, immediately where possible, and, in any event within 7 days after becoming aware of the information. Within 8 days after having initially informed Health Canada of the ADR, submit as complete a report as possible which includes an assessment of the importance and implication of any findings.

Promptly report all serious adverse events that occur at each site along with any adverse events that are both serious and unexpected, and have been reported to Health Canada to your local REB or Clinical Trials Ontario (as applicable).

## 8.3 Monitoring of AEs/SAEs

Any AE that occurs between the times a study participant signs the informed consent form and the time s/he departs the study at the end of the final follow-up visit (or at the time of early discontinuation of the participant from the study for any reason) will be captured and recorded. At each contact with the participant, the investigator (or designate) must seek information on adverse events by specific questioning and, as appropriate, by examination.

All grade 3 AE’s and all non-fatal SAE’s will be followed until resolution or until the site investigator and the clinical/medical monitor, or one of the co-PIs are in agreement that the AE/SAE has stabilized and no more follow-up is required.

## 8.4 Treatment Discontinuation

The criteria for permanent discontinuation of therapeutic or thromboprophylactic anticoagulation in patients allocated to the experimental or standard care arm are as follows:

- Treatment related toxicity (see section 8.0)
- Requirement for prohibited concomitant medications in the therapeutic anticoagulation (experimental arm) (i.e. dual antiplatelet therapy where bleeding risk is deemed unacceptably high by the treating physician, use of any direct oral anticoagulant or any intravenous direct thrombin inhibitor; see Appendix 2)
- Reaching a defined clinical endpoint (i.e. a component of the primary outcome, however, continuation of study treatment is ultimately at the discretion of the treating physician)
- Completion of treatment/intervention as defined by the protocol
- Clinical reasons believed to be life-threatening by the physician, even if not addressed in the toxicity section of the protocol
- Any clinical adverse event, laboratory abnormality, intercurrent illness, other medical condition or situation occurs such that continued anticoagulation would not be in the best interest of the participant.

In order to capture accurate prognostic and safety data, the participant will continue to be followed with the participant’s permission if the study treatment/intervention is discontinued, **and this would not be considered premature study withdrawal (see section 8.5).** There will be no changes to the follow-up visit schedule, except no study treatment/intervention will be administered.

In the event that the participant’s anticoagulation is discontinued due to an AE or SAE, this must be recorded on the CRF. The participant should be followed and treated by the treating physician until the abnormal parameter or symptom has resolved or stabilized. It is up to the clinician to determine that the AE is either resolved or that it has reached a stable state, after which no further follow-up is necessary. There should also be source documentation to support this determination.

If a patient’s medication is discontinued, they will still be followed for primary and secondary outcomes. Participants may be withdrawn from the study if the Sponsor-Investigator terminates the trial (i.e. if new evidence emerges proving either futility or efficacy of the study drug or potential safety concerns or if drug supply becomes an issue).

## 8.5 Premature Study Withdrawal of an Individual Participant

Premature study withdrawal would occur if data collection is to stop before Day 28. The criteria for premature withdrawal from the study for an individual participant are as follows:

- Request of the participant or their substitute decision maker to discontinue data collection for study purposes

## 8.6 Protocol Violations/Deviations

In the event that an investigator becomes aware of a protocol deviation, it is his/her responsibility to document the deviation and report it to the Study Coordinating Centre and the local REB as per local guidelines.

Protocol Deviation: Any alteration/modification to the REB approved protocol. This can involve deviations involving the recruitment or consent process, the sequence and nature of study procedures, the information recorded on subjects, and the documentation used with study subjects.

Major Deviation: A violation that may impact the patient’s rights, safety or welfare, or that can affect the integrity of the study data. Examples are as follows:

- Failure to obtain informed consent
- Enrolment of a patient not meeting inclusion criteria
- Failure to report a serious adverse event
- Study medication dispensing or dosing error
- Study procedure omitted
- Failure to file REB Annual Renewal and/or Modifications

Minor Deviation: A violation that does not have meaningful impact on the study and does not fall into the foregoing categories. These typically involve only logistical or administrative aspects of the study. Examples are as follows:

- Study procedures conducted out of sequence, but with no impact on patient safety or welfare
- Change in contact information of staff
- The use of the same recruitment materials in a new venue (community vs. local newspaper).

# 9.0 Data Handling and Record Keeping

## 9.1 Data Management Responsibilities

Original subject records (source documents) will be reviewed during the course of the monitoring to verify the accuracy of the CRF’s. This review will be conducted according to sponsor monitoring guidelines. The Investigator(s) institutions(s) will permit trial-related monitoring, audits, REB(s) review and regulatory inspections(s) by providing direct access to source data and documentation.

The medical (hospital/practice) records for each patient should contain information to provide a means by which study data can be verified. Patient information collected electronically will be available for review when the study site is monitored or audited.

All source documents and lab reports must be reviewed by the clinical team and data entry staff, who will ensure that they are accurate and complete. Adverse events must be graded, assessed by severity and causality and reviewed by the site investigator or designee.

Data collection is the responsibility of the research staff at the site under the supervision of the Principle Investigator. All data will be entered directly into REDCap (Research Electronic Data Capture). Data will be entered by research staff into REDCap at the following times: screening, randomization (day 1), day 3, day 7, day 14 and day 28. Data entered will cover the entire 28 day period. During the study the investigator must maintain complete and accurate documentation for the study.

If a participant chooses to withdraw from the study prematurely or is withdrawn by the investigator, efforts should be made to collect all related study data until the last completed visit.

REDCap is a secure, web-based application designed exclusively to support data capture for research studies. REDCap provides: 1) an intuitive interface for data entry (with data validation); 2) 128 bit encryption between the data entry client and the server (https); 3) audit trails for tracking data manipulation and export procedures; 4) automated export procedures for seamless data downloads to common statistical packages (SPSS, SAS, Stata, R); 5) procedures for importing data from external sources; and 6) advanced features, such as branching logic and calculated fields.

REDCap is developed and maintained by a team at Vanderbilt University and licensed free of charge by the Applied Health Research Centre (AHRC). The application and data are housed inside a secure data center located at Unity Health Toronto (St. Michael’s Hospital). The data center is physically secured through limited badge access and security cameras. The REDCap servers and application are maintained by the AHRC. Local support for REDCap is provided by AHRC.

## 9.2 Confidentiality

All participant related information including Case Report Forms, laboratory specimens and etc. will be kept strictly confidential and to the extent permitted by the applicable laws. All records will be kept in a secure, locked location and only research staff will have access to the records. Participants will be identified only by means of a coded number specific to each participant. All computerized databases will identify participants by alpha-numeric codes only, and will be password protected or encrypted.

Upon request, participant records will be made available to the study sponsor, monitoring groups representative of the study sponsor, representatives of a participating pharmaceutical sponsor and applicable regulatory agencies such as Health Canada.

## 9.3 Record Retention

To protect the patient’s confidentiality, all study documentation will be kept in a secure location at the study site in compliance with local SOP’s. All sites are expected to store all study documentation for the legally required time period.

The Investigator will maintain all study records according to the ICH-GCP and applicable Health Canada regulatory requirements. Records will be retained for 25 years, in accordance with applicable Health Canada regulatory requirements. If the Investigator withdraws from the responsibility of keeping the study records, custody must be transferred to a person willing to accept the responsibility and the Sponsor-Investigator notified. The Investigator should ensure that no destruction of medical records occurs without the written approval of the Sponsor-Investigator.

# 10.0 Quality Control and Quality Assurance

## 10.1 Study Monitoring Plan

Remote risk-based monitoring will be conducted by qualified central research personnel in order to ensure (1) that the study is carried out according to ICH-GCP E6(R2) guidelines and applicable regulations, and that study related tasks are  performed by staff who are trained and familiar with the clinical study protocol; (2) that study procedures are carried out according to the protocol, and (3) that the data collected is complete, accurate and collected according to the methods defined in the protocol and in associated operational documents.

The proposed remote monitoring scheme will be composed of:

1. Centralized review of essential study document at all participating sites related to participant protection, such as ICF signature pages, GCP and protocol training records, Delegation log, CV and medical license of investigators and Protocol signature page
2. Targeted source data verification (SDV) of eCRF data on randomly selected participants. Only critical data variables identified through risk assessment which are programmed into the EDC system will be source verified.

The monitoring procedures, frequency and country-specific adaptations will be further outlined in the Monitoring Plan.

## 10.2 Role of the Data Safety Monitoring Board

A Data Safety Monitoring Board is a group of experts who meet periodically to review accumulating data gathered from participants in clinical trials with the purpose of protecting

- the safety of the study participants
- the scientific integrity of the study
- the validity of study results

This study will be monitored by an independent data safety monitoring board (DSMB) including a biostatistician, a hematologist, a general internist and intensive care specialist. The four members will not be listed study investigators. The DSMB will be immediately informed of any serious adverse events (SAEs) which may potentially be study drug related. Other SAEs will be reviewed during regular DSMB meetings.

Interim reports, prepared by the data management team for the study, for review by the DSMB will include data on recruitment, compliance, adverse effects, baseline comparability and treatment comparisons. An agreed upon review package which contains the appropriate data summary by treatment will be provided by the study statistician for the purposes of these reviews. Interim analyses will be confidentially provided to the board during the period of study recruitment (see Section 7.3) to evaluate for unquestionable difference in effect between the two study arms and adverse events requiring additional action or discontinuation of the study. DSMB members will review data only by masked study group (such as A vs B rather than experimental vs control). The DSMB may request unblinded safety data tables, in case of adverse events associated with study drug or procedures.

Serious adverse events, which are of concern to members of the Steering Committee (potentially study drug related) will also be reviewed by the DSMB.

Members of the DSMB will also be responsible for developing terms of reference including clinical stopping rules. If at any point the DSMB considers continuance of the study unacceptable, the steering committee will be immediately notified. Thereafter, the study co-PIs can decide whether or not to modify intake to the trial.

## 10.3 Ethics and Research Governance:

This study will be conducted according to Canadian and international standards of Good Clinical Practice and the Health Canada, Food and Drug Act, Part C, Division 5, Drugs for clinical Trials Involving Human Subjects. The Research Ethics Board (REB) policies and standard operating procedures will also be followed.

This protocol and any amendments will be submitted to the REB for formal approval to conduct the study. The decision of the REB concerning the conduct of the study will be made in writing to the investigator.

All participants for this study will be provided a consent form, describing this study and providing sufficient information for participants to make an informed decision about their participation in this study. This consent form will be submitted with the protocol for review and approval by the REB. The formal consent of a participant, using the REB-approved consent form, will be obtained before that participant is submitted to any study procedure. REB approval will also be obtained for any protocol amendments and ICF revisions prior to implementing the changes. Any protocol deviations will be reported to the REB within 15 calendar days.

## 10.4 Feasibility

The Applied Health Research Centre (AHRC) at SMH in Toronto will manage and oversee this trial. AHRC is the largest academic research organization in Toronto, and a designated Research Centre of the Ontario SPOR Support Unit (OSSU) deemed suitable to provide the enabling infrastructure, scientific knowledge and technical support required to conduct patient-oriented research.

## 10.5 Study Product Supply and Accountability

All study medications will be stored at room temperature (as per the labelling) in a secure location. This is an open label study thus once a participant is randomized, a research coordinator will document the treatment allocation in the case report form and notify the MRP and study team by email and phone of the patient’s study treatment allocation (therapeutic or standard care arm). The MRP will then order the study drug in the patient’s medical chart and notify the study team. The research coordinator will then email or fax research pharmacy the order. Research pharmacy will follow their SOP to dispense study drug and study drug will be dispensed via research pharmacy to the treating medical team. Returned and unused study drug will be returned to the pharmacy department where it will be destroyed. Research Pharmacy will maintain drug accountability logs, as per their SOPs.

The study medication should be stored in a secure area according to local regulations. The investigator is responsible for ensuring that it is dispensed only to study participants and only from official study sites by authorized personnel, as dictated by local regulations.

The site investigator is responsible for ensuring that the study medication is stored under the appropriate environmental conditions (temperature, light and humidity).

# 11.0 Study Significance

We believe that this RCT has the potential to **immediately** impact the clinical care of patients with severe COVID-19, internationally, whether our findings are positive or negative as COVID-19 coagulopathy appears to be a highly prevalent complication of severe COVID-19 and may even precede the respiratory manifestations that characterize it (9,10).

In the midst of the COVID-19 pandemic and secondary healthcare crisis, it is time, now more than ever, for randomized care of agents with sound biological plausibility rather than random, best-guess care (22). Thus, our study of a **well understood therapy** with a **predictable adverse effect profile** will, irrespective of our findings, lead to subsequent patients receiving evidence-based therapies and spared from unnecessary harm. Moreover, we are also well positioned to evaluate for ethnic/racial disparities in coagulation/inflammatory responses and effect of anticoagulation.

LMWH and UFH are both licensed anticoagulants in North America therefore, we anticipate swift approval which means that we could **feasibly** launch this RCT at multiple centers **promptly** which is crucially required given the worrisome mortality associated with severe COVID-19 that is rapidly overwhelming hospitals.

# 12.0 Disclosure and Publication Policy

## 12.1 Publication of Study Results

Following completion of the study, the lead Principal Investigators are expected to publish the results of the primary and secondary analyses from this trial, in peer-reviewed scientific journals. A detailed authorship policy will be developed and agreed upon by all investigators to determine how best to fairly acknowledge the contributions of relevant parties.

## 12.2 Data Sharing for Secondary Research

Data from this study may be used for secondary research. All of the individual participant data collected during the trial will be made available after de-identification through expert determination. These data will be made available as soon as possible following publication, with no end date, as part of data sharing requirements from journals and funding agencies, and in the spirit of open data access.

## 12.3 Conflict of interest

The independence of this study from any actual or perceived influence, such as by the pharmaceutical industry, is critical. Therefore, any actual conflict of interest of persons who have a role in the design, conduct, analysis, publication, or any aspect of this trial will be disclosed and managed. Furthermore, persons who have a perceived conflict of interest will be required to have such conflicts managed in a way that is appropriate to their participation in the design and conduct of this trial.

# Table 5. Study Schedule of Events

|  | **Screening^1,2,^** ^$^ | **Randomization^1,2,^**^$^ **(Day 1)** | **Day 3**^$^ | **Day 7**^$^ | **Day 14**^$^ | **Day 28**^$^ |
| --- | --- | --- | --- | --- | --- | --- |
| Informed Consent | X |  |  |  |  |  |
| Inclusion/Exclusion | X |  |  |  |  |  |
| Lowest oxygen saturation (%) on room air | X |  |  |  |  |  |
| Maximum supplemental oxygen (FiO2)^ß^ | X* | X* | X* | X* | X* | X* |
| Concomitant Medications (including other COVID-19 trial treatments) | X* | X* | X* | X* | X* | X* |
| Demographics (age, sex, race/ethnicity^φ^) | X |  |  |  |  |  |
| Medical History (including smoking, date of COVID-19 symptom onset) | X* |  |  |  |  |  |
| Height | X** |  |  |  |  |  |
| Weight | X** |  |  |  |  |  |
| Dose and frequency of therapeutic anticoagulation administered (treatment arm) |  | X* | X* | X* | X* | X* |
| Dose and frequency of LMWH or UFH at thromboprophylactic doses (standard care arm) |  | X* | X* | X* | X* | X* |
| Complete Blood Count |  | X* | X* | X* | X* | X* |
| Ferritin |  | X* | X* | X* | X* | X* |
| D-Dimer | X* | X* | X*** | X* | X* | X* |
| PT |  | X* | X* | X* | X* | X* |
| aPTT^3^ |  | X* | X* | X* | X* | X* |
| Anti-Xa for UFH^3^ |  | X* | X* | X* | X* | X* |
| Anti-Xa for LMWH |  | X* | X* | X* | X* | X* |
| INR |  | X* | X* | X* | X* | X* |
| Fibrinogen |  | X* | X* | X* | X* | X* |
| Lactate dehydrogenase |  | X* | X* | X* | X* | X* |
| Troponin |  | X* | X* | X* | X* | X* |
| C-Reactive Protein |  | X* | X* | X* | X* | X* |
| Creatinine |  | X* | X* | X* | X* | X* |
| ALT |  | X* | X* | X* | X* | X* |
| Heparin-Platelet Factor 4 Immunoassay |  | X* | X* | X* | X* | X* |
| Blood group |  | X^∞^ | X^∞^ | X^∞^ | X^∞^ | X^∞^ |
| Primary outcome assessment^4^ |  | X* | X* | X* | X* | X* |
| Length of Hospital Stay |  | X* | X* | X* | X* | X* |
| Bleeding Event^5^ |  | X* | X* | X* | X* | X* |
| Red Blood Cell transfusion^6a^ and/or transfusion of any hemostatic blood product/component^6b^ |  | X* | X* | X* | X* | X* |
| Renal replacement therapy^7^ |  | X* | X* | X* | X* | X* |
| Organ support (see Appendix 4) |  | X* | X* | X* | X* | X* |
| Venous or arterial thromboembolism^8^ |  | X* | X* | X* | X* | X* |
| AE/ SAE Assessment^9^ |  | X* | X* | X* | X* | X* |
| Optional Biobanking |  | X† | X† |  | X† |  |

* collected through standard of care when available in the chart;

** height and weight to be collected from the patient’s chart or obtained verbally from the patient;

*** D-dimer will be drawn once on day 2 after randomization (+/- 24 hours); if collected through standard of care, study specific D-Dimer is not needed);

^ß^See Appendix 3 for FiO2 for nasal cannula and Venturi mask;

^φ^Race/ethnicity of participant collected given known ethnic variation of D-dimers (categories defined in Appendix 1 (23); to obtain verbally from the patient);

^∞^to be obtained from hospital medical record or verbally from patient if not available from medical record and patient is aware of their blood group;

†Phlebotomy and sample processing on day 1, 3, and 14 OR the time of discharge OR reaching a component of primary outcome OR study withdrawal for any reason;

^$^Research staff will enter data into REDCap on screening, randomization (day 1), Days 3, 7, 14, and 28 including data from the days in between. Data entered will cover the entire 28 day period. Day 3 will cover Day 2 to Day 3, Day 7 will cover Day 4 to Day 7, Day 14 will cover Day 8 to Day 14, and Day 28 will cover Day 15 to Day 28. If multiple laboratory results are available, the most pathological value since last assessment will be recorded;

^1^within 5 days (i.e. 120 hours) of hospital admission;

^2^screening and randomization can be done on the same day;

^3^ For participants treated with UFH: aPTT or UFH anti-Xa will be drawn according to local institutional UFH nomogram protocol guidance;

^4^If patient is discharged alive before 28 days, vital status will be determined using telephone follow-up by a research coordinator; the use of BIPAP or CPAP at night or when sleeping for sleep apnea is not considered non-invasive mechanical ventilation or organ support for the purpose of this trial; If a patient was discharged alive on mechanical ventilation (invasive or non-invasive) prior to day 28, a call to the patient or a doctor/nurse from the rehabilitation health facility is needed to confirm ventilation status on day 28 and their last day of mechanical ventilation.
^5^Bleeding defined as by ISTH Scientific and Standardization Committee (ISTH-SSC) recommendation: 1) Fatal bleeding, and/or 2) symptomatic bleeding in critical area or organ (such as intracranial, intraspinal, intraocular, retroperitoneal, intraarticular or pericardial, or intramuscular with compartment syndrome, and/or 3) bleeding causing a fall of hemoglobin level of 20g/L or more, or leading to transfusion of two or more units of whole blood or red cells; ^6a^Defined as >1 unit;

^6b^Defined as platelet, frozen plasma, prothrombin complex concentrate, cryoprecipitate, fibrinogen concentrate;

^7^defined as continuous renal replacement therapy or intermittent hemodialysis;
^8^defined via diagnostic imaging and/or electrocardiogram where appropriate and possible; ^9^AE/SAE will be recorded on any day.

# 13.0 References

1. Guan W, Ni Z, Hu Y, Liang W, Ou C, He J, et al. Clinical Characteristics of Coronavirus Disease 2019 in China. N Engl J Med. 2020 Feb 28;NEJMoa2002032.

2. Tang N, Bai H, Chen X, Gong J, Li D, Sun Z. Anticoagulant treatment is associated with decreased mortality in severe coronavirus disease 2019 patients with coagulopathy. J Thromb Haemost [Internet]. 2020 Mar 27 [cited 2020 Mar 31]; Available from: http://doi.wiley.com/10.1111/jth.14817

3. Tang N, Li D, Wang X, Sun Z. Abnormal coagulation parameters are associated with poor prognosis in patients with novel coronavirus pneumonia. J Thromb Haemost. 2020 Apr;18(4):844–7.

4. Thachil J, Tang N, Gando S, Falanga A, Cattaneo M, Levi M, et al. ISTH interim guidance on recognition and management of coagulopathy in COVID-19. J Thromb Haemost [Internet]. 2020 Mar 25 [cited 2020 Mar 31]; Available from: http://doi.wiley.com/10.1111/jth.14810

5. Zhou F, Yu T, Du R, Fan G, Liu Y, Liu Z, et al. Clinical course and risk factors for mortality of adult inpatients with COVID-19 in Wuhan, China: a retrospective cohort study. The Lancet. 2020 Mar;395(10229):1054–62.

6. Huang C, Wang Y, Li X, Ren L, Zhao J, Hu Y, et al. Clinical features of patients infected with 2019 novel coronavirus in Wuhan, China. The Lancet. 2020 Feb;395(10223):497–506.

7. Lippi G, Plebani M, Henry BM. Thrombocytopenia is associated with severe coronavirus disease 2019 (COVID-19) infections: A meta-analysis. Clin Chim Acta. 2020 Jul;506:145–8.

8. Luo W, Yu H, Gou J, Li X, Sun Y, Li J, et al. Clinical Pathology of Critical Patient with Novel Coronavirus Pneumonia (COVID-19). 2020 Mar 9 [cited 2020 Mar 31]; Available from: https://www.preprints.org/manuscript/202002.0407/v4

9. Li L, Ma X, Li X. [Effect of heparin on histone-mediated the expression of von Willebrand factor and fibrinogen in lung tissue]. Zhonghua Wei Zhong Bing Ji Jiu Yi Xue. 2019 Nov;31(11):1363–7.

10. Mu S, Liu Y, Jiang J, Ding R, Li X, Li X, et al. Unfractionated heparin ameliorates pulmonary microvascular endothelial barrier dysfunction via microtubule stabilization in acute lung injury. Respir Res. 2018 Nov 15;19(1):220.

11. Klok FA, Kruip MJHA, van der Meer NJM, Arbous MS, Gommers DAMPJ, Kant KM, et al. Incidence of thrombotic complications in critically ill ICU patients with COVID-19. Thromb Res [Internet]. 2020 Apr 10 [cited 2020 Apr 10]; Available from: http://www.sciencedirect.com/science/article/pii/S0049384820301201

12. Zhang B, Zhou X, Qiu Y, Feng F, Feng J, Jia Y, et al. Clinical characteristics of 82 death cases with COVID-19. medRxiv. 2020 Jan 1;2020.02.26.20028191.

13. Stephanie Lee, Michael Fralick, Brandon Tse, Grace Tang, Michelle Sholzberg. COVID-19 and coagulopathy:a Systematic Review [COVID-COAG]. [Internet]. [cited 2020 Apr 9]. Available from: https://www.crd.york.ac.uk/prospero/display_record.php?RecordID=175871

14. Shanghai Expert Group on Clinical Treatment of New Coronavirus Diseases. Expert Consensus on Comprehensive Treatment of Coronavirus Diseases in Shanghai in 2019. Chin J Infect Dis.

15. Raffield Laura M., Zakai Neil A., Duan Qing, Laurie Cecelia, Smith Joshua D., Irvin Marguerite R., et al. D-Dimer in African Americans. Arterioscler Thromb Vasc Biol. 2017 Nov 1;37(11):2220–7.

16. Bikdeli B, Madhavan MV, Jimenez D, Chuich T, Dreyfus I, Driggin E, et al. COVID-19 and Thrombotic or Thromboembolic Disease: Implications for Prevention, Antithrombotic Therapy, and Follow-up. J Am Coll Cardiol [Internet]. 2020 Apr 17 [cited 2020 Apr 17]; Available from: http://www.onlinejacc.org/content/early/2020/04/15/j.jacc.2020.04.031

17. Wood L, Egger M, Gluud LL, Schulz KF, Jüni P, Altman DG, et al. Empirical evidence of bias in treatment effect estimates in controlled trials with different interventions and outcomes: meta-epidemiological study. BMJ. 2008 Mar 15;336(7644):601–5.

18. Pfizer. Fragmin Product Monograph [Internet]. Available from: https://www.pfizer.ca/sites/default/files/201810/FRAGMIN_PM_E_210778_18Oct2018.pdf

19. Sanofi-aventis Canada. rLOVENOX® Product Monograph. http://products.sanofi.ca/en/lovenox.pdf.

20. LEO Pharma. PRODUCT MONOGRAPH - Pr innohep® [Internet]. Available from: https://www.leo-pharma.ca/Files/Filer/LEO_local_downloads/LEO-Pharma.ca/innohep_PM_(8.0)_-_26-MAY-2017[1].pdf

21. Schulman S, Kearon C. Definition of major bleeding in clinical investigations of antihemostatic medicinal products in non-surgical patients. J Thromb Haemost. 2005;3(4):692–4.

22. Cheng MP, Lee TCL, Tan DHS, Murthy S. Generating randomized trial evidence to optimize treatment in the COVID-19 pandemic. CMAJ [Internet]. 2020 Jan 1 [cited 2020 Apr 1]; Available from: https://www.cmaj.ca/content/early/2020/03/26/cmaj.200438

23. NOT-OD-15-089: Racial and Ethnic Categories and Definitions for NIH Diversity Programs and for Other Reporting Purposes [Internet]. [cited 2020 Apr 17]. Available from: https://grants.nih.gov/grants/guide/notice-files/NOT-OD-15-089.html

# Appendix 1. Racial and Ethnic Categories and Definitions

**Definitions for Racial and Ethnic Categories**

(based on NIH categories that were modified to be inclusive of additional categories defined at <http://torontohealthequity.ca/wp-content/uploads/2017/05/TC-LHIN-Hospitals-Demographic-Questions-English-visible-v2-1.pdf)>

- **American Indian, Alaska Native, First Nations, Indigenous/Aboriginal, Metis.** A person having origins in any of the original peoples of North and South America (including Central America), and who maintains tribal affiliation or community attachment.
- **Asian.** A person having origins in any of the original peoples of the Far East, Southeast Asia, or the Indian subcontinent including, for example, Cambodia, China, India, Japan, Korea, Malaysia, Pakistan, the Philippine Islands, Thailand, and Vietnam.
- **Black or African American.** A person having origins in any of the black racial groups of Africa. Terms such as "Haitian" or "Negro" can be used in addition to "Black or African American."
- **Hispanic or Latino.** A person of Cuban, Mexican, Puerto Rican, South or Central American, or other Spanish culture or origin, regardless of race. The term, "Spanish origin," can be used in addition to "Hispanic or Latino."
- **Native Hawaiian or Other Pacific Islander.** A person having origins in any of the original peoples of Hawaii, Guam, Samoa, or other Pacific Islands.
- **White.** A person having origins in any of the original peoples of Europe, the Middle East, or North Africa.

The categories and definitions provide a common language to promote uniformity and comparability of data on race and ethnicity.  Moreover, federal agencies have a continuing commitment to monitor the operation of its review and award processes to detect, and deal appropriately with, any instances of real or apparent inequities.  All analyses conducted on race and ethnicity report aggregate statistical findings and do not identify individuals.

# Appendix 2. Requirement for prohibited concomitant medications in the therapeutic anticoagulation (experimental arm)

**Dual therapy with any of the following:**

Aspirin

Clopidogrel

Ticagrelor

Prasugrel

**Single agent therapy with any of the following:**

Dabigatran

Rivaroxaban

Apixaban

Edoxaban

Melagatran

Inogatran

Argatroban

Bivalirudin

Lepirudin

Desirudin

Hirudin

Alteplase

Streptokinase

Urokinase

Reteplase

Tenecteplase

# Appendix 3. Fraction of Inspired Oxygen (FiO2) for Various Oxygen Delivery Devices

For all supplemental oxygen delivery devices, the patient is not just breathing the direct oxygen, but rather is breathing a **combination** of room air plus the oxygen from the supplemental device. Different devices deliver to the patient more or less of a % of what is coming in from the tank.

| 0 (no oxygen, just room air) | 21% |
| --- | --- |

**For a Nasal Cannula:**

| Oxygen tank **FLOW RATE** in liters / min | **FiO2** -- Fraction of Inspired Oxygen value |
| --- | --- |
| 1 L / min | 24% |
| 2 L / min | 28% |
| 3 L / min | 32% |
| 4 L / min | 36% |
| 5 L / min | 40% |
| 6 L / min | 44% |

**For a Simple Face Mask – Low Flow (approximate values):**

| Oxygen tank **FLOW RATE** in liters / min | **FiO2** -- Fraction of Inspired Oxygen value |
| --- | --- |
| 5 L / min | 35% |
| 6 L / min | 39% |
| 7 L / min | 43% |
| 8 L/min | 47% |
| 9 L/min | 51% |
| 10 L/min | 55% |

**For a Venturi Mask (approximate values):**

The FiO2 is controlled by both the L/min as well as by the valve as indicated by its colour (see below). Note colours may vary based on institution (please double check locally). See example below, the colour of the valve is the best indicator of the FiO2 provided.


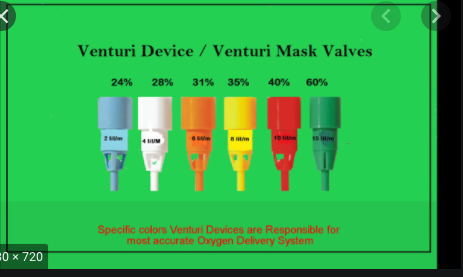


**blue white orange yellow red green**

**Nonrebreather (approximate value):**

| Oxygen tank **FLOW RATE** in liters / min | **FiO2** -- Fraction of Inspired Oxygen value |
| --- | --- |
| 15 L/min | 90% |

**High Flow Nasal Cannula:**

**For the purposes of this trial, a patient is considered to be receiving high flow nasal cannula (HFNC) therapy when the flow rates provided by this device are >30 L/min.**

For HFNC therapy, the FiO2 and flow rate are independent settings.

Thus, you cannot infer the FiO2 based on the flow rate.

The user controls both and thus should simply enter the FiO2 that was set for the patient.

# Appendix 4. Outcome Definition for Organ-support Free Days

**Outcome Definition for Organ-support Free Days**

Defined as the number of days that a patient is alive and free of organ support through 28 days after trial entry. Organ support is defined as receipt of non-invasive mechanical ventilation, high flow nasal cannula oxygen, invasive mechanical ventilation, or vasopressor therapy.

- Non-invasive mechanical ventilation is defined as BIPAP or CPAP when used for acute respiratory support (the use of BIPAP or CPAP at night or when sleeping for sleep apnea is not considered organ support).
- High Flow Nasal Cannula Oxygen is defined as receiving ≥ 30 l/min flow at FiO2 ≥ 40%.
- Invasive mechanical ventilation is defined as positive pressure ventilation through endotracheal tube or tracheostomy.
- Vasopressor support includes infusion of any vasoactive or inotropic medication.
- A patient must be extubated and not receiving mechanical ventilation for at least 2 days before being considered free of mechanical ventilation. If a patient was extubated and re-intubated and placed back on mechanical ventilation within 1 or 2 days, the patient is considered to be on mechanical ventilation during those 1 or 2 days before re-intubation.
- Any patient dying in the acute hospital stay are assigned 28 Day Organ-Support Free Days of –1.
- If there is intervening time in which a patient is free of organ support but goes back on organ support the intervening time does not count toward the organ support free days endpoint. Only time before organ support and after the last use of organ support are counted as “free days.”
- If a patient was discharged alive without mechanical ventilation prior to Day 28, the patient is assumed to be free of organ support after hospital discharge for the remainder of the 28 days.
- If a patient was discharged alive on mechanical ventilation (invasive or non-invasive) prior to day 28, a call to the patient or a doctor/nurse from the rehabilitation health facility is needed to confirm ventilation status on day 28 and their last day of mechanical ventilation.
